# Hybrid trial for Alcohol reduction among people with TB and HIV in India (HATHI): Protocol for a Hybrid Type 1 Effectiveness-Implementation Randomized Controlled Trial

**DOI:** 10.21203/rs.3.rs-8759034/v1

**Published:** 2026-03-25

**Authors:** Geetanjali Chander, Nishi Suryavanshi, Nikhil Gupte, Gauri Dhumal, Shatabdi Bagchi, Rohidas Borse, Arjun Lal Kakrani, Abhijit Nadkarni, Sohn Hojoon, Heidi Hutton, Amita Gupta

**Affiliations:** University of Washington; Johns Hopkins Centre for Infectious Diseases in India; Johns Hopkins Centre for Infectious Diseases in India; Johns Hopkins Centre for Infectious Diseases in India; Johns Hopkins Centre for Infectious Diseases in India; Byramjee Jeejeebhoy Government Medical College/Sassoon General Hospital; Dr. D.Y. Patil Medical College, Hospital and Research Centre; London School of Hygiene and Tropical Medicine; Seoul National University; Johns Hopkins University; Johns Hopkins University

**Keywords:** TB, HIV, alcohol, India, brief intervention, randomized controlled trial

## Abstract

**Background:**

The highest incidence of tuberculosis disease (TB) in the world is in India, accounting for 26% of all new cases globally, with approximately 48,000 among persons with HIV (PWH). Unhealthy alcohol use can worsen the health of people who have TB or HIV and those who have both TB and HIV. Behavioral interventions that target alcohol use and are integrated into TB and HIV care may lead to better outcomes. This paper describes the protocol for HATHI, **H**ybrid trial for **A**lcohol reduction among people with **T**B and **H**IV in **I**ndia, a Hybrid Type 1 effectiveness-implementation trial, that tests if a behavioral intervention integrated into TB and HIV treatment, compared to usual care, results in lower alcohol use and improved TB and HIV health outcomes among people with unhealthy alcohol use in Pune, India.

**Methods:**

HATHI is a randomized controlled trial that will recruit 450 people with unhealthy alcohol use from TB and HIV clinics in Pune, India. The aims of HATHI are to: (1) test if a four session behavioral intervention integrated into TB and HIV care results in lower alcohol use among persons with TB, HIV and TB/HIV coinfection compared to usual care as measured by phosphatidyl ethanol (PEth), an alcohol biomarker (primary outcome); (2) test if the same intervention also leads to improved TB and HIV clinical outcomes including TB and HIV medication adherence, HIV viral suppression, TB sputum/culture conversion and the composite outcome of TB treatment failure, default or death; (3) evaluate barriers and facilitators to integrating the intervention into TB and HIV care, and (4) determine the incremental costs of delivering the intervention in these clinical settings. We hypothesize that the HATHI intervention will result in lower alcohol use compared to standard of care use at 6 months among people and superior TB and HIV clinical outcomes at 12 months.

**Discussion:**

This intervention addresses unhealthy alcohol use, a known barrier to optimal TB and HIV treatment outcomes. If HATHI proves effective, insights into barriers and facilitators for integration will inform future scale-up of this behavioral alcohol intervention in TB and HIV clinical settings.

**Trial registration:**

ClinicalTrials.gov NCT04230395 (first submission 1/18/2020, most recently update 2/17/25) and India Clinical trial Registry CTRI/2020/03/024141

## Background and Rationale

India has the highest incidence of TB in the world, accounting for 26% of all new cases globally, with 48,000 among People With HIV (PWH).(1, 2)  India also ranks third globally in absolute HIV burden, with 2.6 million PWH and 64,000 new HIV infections in 2024.(3) PWH are 14–18 times more likely to develop TB, and TB is the leading cause of death among PWH.(4) Unhealthy alcohol use encompassing heavy use, heavy episodic (binge) drinking, and alcohol use disorders (AUD),(5) triples the risk of TB in the general population, increasing susceptibility to primary infection and reactivation, and also leads to poor outcomes including decreased treatment adherence, treatment default, treatment failure, relapse, and death.(6–10)

In India, alcohol use is on the rise.(11, 12) Our prior work in India has demonstrated 1) a high prevalence of unhealthy alcohol use among patients with TB, and 2) that unhealthy alcohol use is one of the major reasons for treatment default, failure, and mortality.(9, 10, 13, 14) Among PWH, unhealthy alcohol use is associated with HIV transmission, decreased use of and adherence to ART, lower viral suppression, decreased engagement and retention in care, more rapid HIV disease progression, and mortality.(15–21)

Individual-level behavioral alcohol interventions have been associated with significantly decreased HIV viral loads and increased medication adherence among PWH.(22) Such interventions among persons with TB show promise among people with TB but data are limited.(14, 23, 24) Further, despite the high burden of unhealthy alcohol use, behavioral interventions are not commonly available in TB and HIV care settings.

Brief, two to four session alcohol interventions, consisting of Cognitive Behavioral Therapy (CBT) and Motivational Enhancement Therapy (MET) are the most widely used and empirically supported behavioral interventions to reduce unhealthy alcohol use and alcohol-related harms and most frequently culturally adapted for low and middle income county (LMIC) use.(25–30) We have developed and tested culturally relevant brief counseling interventions for unhealthy alcohol use among PWH in Vietnam,(31) and for primary care settings in India.(32, 33) Maintaining the core components of these brief alcohol interventions, we have adapted them for use in HIV and TB clinical settings in India and now seek to test its effectiveness.

### Objectives

In this manuscript, we describe the protocol for HATHI, **H**ybrid trial for **A**lcohol reduction among people with **T**B and **H**IV in **I**ndia**).** Using a two arm, parallel group randomized controlled trial (RCT) design, this Hybrid Type 1 Implementation Effectiveness study tests the effectiveness of a behavioral counseling intervention (BI) on alcohol reduction (primary), and TB and HIV clinical outcomes (secondary) compared to usual care in TB and HIV clinical settings. Our aims are as follows:

#### Aim 1:

To examine the effectiveness of BI integrated into TB and HIV care compared to usual care on alcohol use.

#### Aim 2:

To examine the effectiveness of BI integrated into TB and HIV care compared to usual care on TB and HIV treatment outcomes.

#### Aim 3:

Guided by the Reach, Effectiveness, Adoption, Implementation, Maintenance (RE-AIM) implementation framework(34, 35), and using mixed methods, to 3a) evaluate patient, provider and organizational barriers and facilitators to integrated BI in TB and HIV settings

#### Aim 4:

To measure incremental costs from health system and societal perspectives of integrated BI in TB and HIV settings.

We hypothesize that compared to usual care, BI will result in 1) lower alcohol use at six months (primary outcome), 2) decreased TB treatment default, failure or death, 3) increased TB medication adherence, 4) increased retention in TB care; and among PWH, 5) increased HIV-RNA suppression, 6) increased ART adherence, and 7) increased retention in HIV care.

### Trial Design

**Figure 1:**
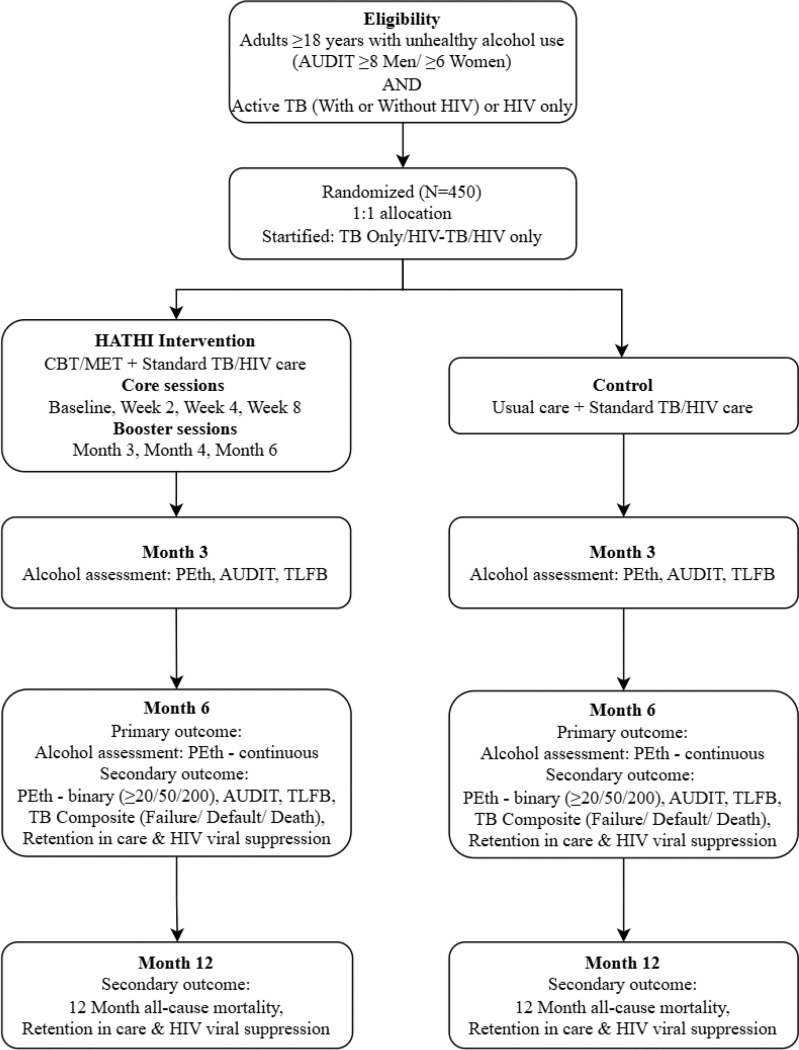
HATHI trial design

HATHI is an on-going Hybrid Type 1 implementation effectiveness study with individual level randomization to intervention or usual care in a 1:1 ratio. A Hybrid Type 1 design, defined by Curran and colleagues as a study that primarily tests effectiveness of a clinical intervention and secondarily gathers information on its implementation potential,(36, 37) was chosen to increase efficiency of implementation should the intervention be effective. Usual care was chosen as the control condition because BI has not been tested for PWH and people with TB in India, and therefore its effectiveness in this setting has not been established. Figure 1 depicts the overall trial design.

## Methods: Participants, Interventions and Outcomes

### Study setting

The trial and all associated procedures will take place at two sites in India: Byramjee Jeejeebhoy Government Medical College (BJGMC) which is associated with Sassoon General Hospital, a major public tertiary care center (BJGMC-SGH), and Dr. D.Y. Patil Medical College and Research Centre (DYPMC), a major private tertiary care center, both in Pune, India. BJGMC is a hub for medical research, collaborating with institutions worldwide, and is in Pune Municipal Corporation Area (pop. 6.7 million). Its 1800-bed public hospital cares for underserved populations from surrounding urban and rural communities. It houses one of India’s largest HIV public sector care centers funded by India’s National AIDS Control Organization and Maharashtra State AIDS Control Society; > 24,000 PWH have been registered since 2005, and currently > 5000 patients are on antiretroviral therapy (ART). Approximately 50 PWH are seen daily with 1–2 new patients per day. The TB/Chest Department has an outpatient clinic and a 65-bed inpatient ward where 75–100 outpatients with TB and 10–15 inpatient TB admissions are evaluated daily. There are 30–35 new TB diagnoses per month among people without HIV and 12–15 new TB cases per month among PWH. DYPMC is in the Pimpri-Chinchwad Municipal Corporation area of Pune district. It has an affiliated 1470-bed hospital that serves a population of 3+ million. Like BJGMC, DYPMC has a public HIV Integrated Counselling and Testing Centre and a linked ART clinic at Yashwantrao Chavan Memorial Hospital funded by the Indian Government. Approximately 10–15 new PWH are registered per month. The Department of Respiratory Medicine has an outpatient clinic and a 60-bed inpatient ward and 7 bed ICU. They evaluate 150 outpatients for TB monthly of which 25–30 are newly diagnosed with TB. Approximately 20–25 PWH with or without TB are seen per month. This trial will be implemented in collaboration with the Maharashtra state and local TB programs. Prior to study funding, patients, caregivers and providers were engaged to discuss how to optimally integrate alcohol screening into these clinical practices and where the intervention should be delivered, thereby informing clinic based intervention instead of home-based intervention.

### Eligibility Criteria

Inclusion Criteria:

- Unhealthy alcohol use defined as Alcohol Use Disorders Identification Test (AUDIT) Score ≥ 8 in men / ≥ 6 in women;
- Age ≥ 18 years of age;
- Active TB (with or without HIV) defined as either:
  - a) Microbiologically confirmed TB (sputum AFB smear positive by microscopy or +GeneXpert at entry) or
  - b) Clinical TB that is subsequently confirmed by Acid-Fast Bacilli (AFB) culture;
- Initiating TB therapy within 30 days of enrollment.

OR

- Unhealthy alcohol use defined as AUDIT Score ≥ 8 in men / ≥ 6 in women;
- Age ≥ 18 years of age;
- HIV infection.

Exclusion Criteria:

- In current treatment for unhealthy alcohol use;
- Unable to participate in intervention sessions either due to severity of medical illness, cognitive dysfunction or active psychosis;
- Pregnant (will refer directly to alcohol treatment);
- Household member of current study participant;
- TB Cohort only: Patients reported to have drug-resistant TB, multidrug-resistant TB (MDR-TB), or extrapulmonary TB (EPTB). (Note: People with HIV and EPTB are not excluded)

Eligible participants with 1) severe alcohol use disorder, determined by the Mini International Neuropsychiatric Interview (MINI) MINI DSM-V,(38) 2) a history of alcohol withdrawal, or 3) a Clinical Institute Withdrawal Assessment (CIWA)(39) score of 12 or greater, will first be evaluated by psychiatry faculty at study sites, BJGMC or DYPMC for medical clearance prior to enrollment due to risk of alcohol withdrawal.

### Interventions:

#### Usual Care (UC):

Individuals randomized to the UC arm will receive provider advice to reduce or abstain from alcohol use and referral to alcohol treatment services at the provider’s discretion. In addition to usual care, at all visits, participants will also receive vital signs as part of the study to monitor blood pressure and pulse, and the CIWA will be performed to screen for alcohol withdrawal.

All participants, irrespective of assigned study condition, will receive standard of care for TB and HIV treatment, including 4-drug TB therapy and for PWH, ART. Individuals with TB randomized to UC arm will return for their routine TB treatment visits at weeks 4 and 8 where sputum will be collected per standard of care. They will have an additional study visit at week 2, where sputum will be obtained. PWH will receive standard of care delivered by the HIV clinic which includes monthly monitoring of ART side effects, clinical assessment, TB and other opportunistic infection evaluations, counseling for psychosocial and adherence support, CD4 count every 6 months, and viral load testing every 12 months.

### Intervention

Individuals randomized to the intervention will receive UC plus the HATHI intervention. The **HATHI intervention** is a manualized behavioral intervention that integrates evidence-based components of CBT/MET for alcohol reduction. The content intervention was, systematically adapted for delivery within Indian TB and HIV clinical settings and provides a counselor script to aid consistency and quality of counselor delivery. The intervention comprises four core sessions of approximately 45 minutes each, supplemented by three booster sessions of 10–15 minutes, delivered over a three-month period (at month 3, 4 and 6) and aligned to regular treatment visits when possible. The number of boosters was selected to take advantage of monthly scheduled visit during the 6 months of TB therapy.

HATHI is designed to provide relevant alcohol and health information, enhance motivation, strengthen self-efficacy, and teach practical skills to reduce at-risk alcohol use. Sessions are delivered in a **Motivational Interviewing–informed style**, emphasizing collaboration and client autonomy while encouraging individuals to explore their own reasons for change within a supportive, nonjudgmental context. Table 1 describes session timetable and content.

### Intervention Supervision and fidelity monitoring

Co-investigators (SB and GD) will initially provide weekly supervision and then transition to monthly frequency and periodically conduct direct observation of counseling sessions to ensure protocols are followed properly. Peer supervision will occur weekly throughout the study period. All the intervention sessions will be audio recorded. Initially, the first five audio recordings of the sessions of each counselor, and later a random subset of 15% of recorded sessions will be assessed to ensure fidelity to the intervention manual.

The intervention sessions will be supervised using the Motivational Interviewing Coach Rating Scale (MI-CRS).(40, 41) The purpose of the supervision is to improve counselors’ MI skills and ensure better client outcomes. MI-CRS assesses counselor skills across 12 criteria, which include empathy, collaboration, open-ended questioning, affirmation, reflective listening, summarizing, evoking client motivations, respecting client autonomy in goal setting, and balancing the client’s agenda with a focus on target behaviors. Each criterion is scored on a scale from one to four, corresponding to skill levels: beginner (1), novice (2), intermediate (3), and advanced (4).

Fidelity monitoring: In addition, the MI-CRS will be used to measure fidelity to the motivational interviewing style in intervention delivery.(40, 41) Supervisors (SB, GD) completed six hours on online MI-CRS training (three hours per day over two days), eight hours of online MI training (four hours per day over two days), and 14 days of on-site training with the study staff at the Pune office. Reliability is checked every month by (SB and GD) co-rating 1–2 sessions for adherence to MI and BI session content.

BI session supervision updates, challenges and fidelity issues encountered during implementation will be discussed in bi-monthly calls with HH, a clinical psychologist and co-investigator. These meetings will focus on reviewing session feedback and identifying strategies for improvement.

Bi-monthly reports are shared with the principal investigators, detailing session information and ratings on the MI-CRS, along with qualitative feedback for counselors that highlights their strengths and areas for improvement. Number of intervention sessions attended and booster sessions delivered will be captured in the REDCap database.

### Criteria for discontinuing or modifying allocated interventions

The intervention will be discontinued for people who become incapacitated and unable to participate in counseling visits. We will not exclude participants who return to alcohol use or are stepped up into more intensive treatment services, since these are expected outcomes for this patient population. We will attempt to retain these patients in the protocol, and the counselor will continue efforts at re-engagement unless the participant formally withdraws from the study. In the event that a participant is excluded from the study prematurely, they will continue to receive all services provided by the TB and HIV Clinics and be referred for alcohol treatment services as needed.

### Provisions for post-trial care

Individuals requesting continued alcohol care post-trial will be referred to the substance use outpatient program at BJGMC and DYPMC.

#### Outcomes

Primary outcome (Specific Aim 1): Our primary outcome is alcohol use at six months as measured by the alcohol biomarker Phosphatidyl Ethanol (PEth).(42) PEth will be collected using dried blood spots. In the initial proposal, the primary outcome measure was based on alcohol self-report, using the Time Line Follow Back (TLFB) interview.(43, 44) At the HATHI DSMB meeting on May 30, 2024, the DSMB recommended changing to PEth due to observed under-reporting of alcohol use on the TLFB by participants compared to PEth.

Secondary alcohol outcomes: We will examine the following secondary outcomes of PEth at Month 6and 12:

1. PEth as a binary variable at different thresholds of alcohol use: PEth ≥ 20; ≥50; ≥ 200
2. Total drinking days in the last 30 days measured using TLFB (count)
3. AUDIT score (Cut off: ≥8 for men, ≥ 6 for women), and continuous
4. Composite TLFB and PEth: Total drinking days using TLFB and PEth ≥ 20; ≥50; ≥ 200
5. Composite AUDIT score and PEth: AUDIT ≥ 8 / ≥ 6 men/women & PEth ≥ 20; ≥50; ≥ 200

Other secondary clinical outcomes (Specific Aim 2) are described in Tables 2 and 3 and defined further in Appendix 1.

##### Implementation outcomes (Specific Aim 3)

RE-AIM dimensions (34, 35), and implementation outcomes of feasibility, acceptability, appropriateness, fidelity and sustainability(35, 47) will be quantitatively assessed through study records and surveys, and qualitatively using study team meeting/counselor supervision notes and in-depth interviews focused on perceptions (beliefs, feelings) of the intervention and barriers and facilitators to implementation. Table 4 describes these outcomes.

##### Economic evaluation (Specific Aim 4)

For economic evaluation, we will assess three types of costs: implementation process costs, per-patient service delivery costs, and patient perspective costs. Implementation cost will be defined as cumulative total programmatic cost of the HATHI intervention implementation, categorically analyzed by three phases of implementation: design, initiation, and maintenance phase.(48) HATHI intervention delivery cost will be assessed as per-patient cost, incorporating implementation costs and direct healthcare resources utilized. Patient perspective costs will be assessed as cumulative patient direct out-of-pocket and indirect costs during the participants’ TB treatment until the end of patient follow-up. For cost-effectiveness analysis, the primary outcome will be the incremental cost per alcohol-free patient at endline. Secondary outcomes include incremental cost per improvements in health-related quality of life (HRQoL) score measured using the WHO QoL-BREF(49) and/or EQ-5D.(50) Additionally, we will use the trials’ primary and secondary clinical effectiveness outcomes to estimate modeled long-term projections of health-related quality of life improvements attributable to alcohol reduction and TB treatment outcomes assessed in terms of disability adjusted life years (DALYs) averted.

#### Participant timeline:

**Figure 2:**
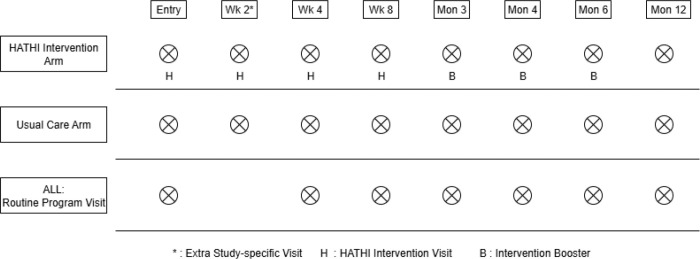
Baseline and follow-up visits by study arm

Figure 2 depicts the participant timeline. Baseline, week 4, week 8, month 3, month 4, month 6 and month 12 visits coincide with routine clinic visits. As part of the clinical trial, there is an extra visit at week 2 where the intervention will be delivered to those randomized to the intervention and both arms will undergo sputum collection.

##### Sample size

The effectiveness of the HATHI intervention will be assessed at 6 months using PEth as the primary outcome. A total sample size of 450 participants provides 80% power at a 5% significance level to detect a minimum mean difference of 90 ng/mL between randomized arms. This calculation assumes a mean PEth of 212 ng/mL (SD = 205 ng/mL), as observed in the ongoing HATHI study. Calculations assume a 10% loss to follow-up, and intra-class correlation coefficient of 0.04 to account for site-counsellor clustering.

#### Recruitment

Participants will be recruited from the HIV and TB clinics at BJGMC and DYPMC and through referrals from peripheral TB & HIV clinics. We will recruit through provider referral. The study team will approach providers in the respective TB and HIV clinics at BJGMC and DYPMC and will request that they refer individuals who endorse alcohol use on the standard National Tuberculosis Elimination Programme patient intake form or ART treatment card. Additionally, study staff will review NTEP registers bi-weekly to identify all potentially eligible participants within 30 days of their TB treatment at BJGMC and DYPMC TB clinics. For eligible participants who decline, reasons will be documented and discussed by study staff. Accrual reports will be reviewed regularly, and additional strategies will be implemented if required to achieve the target sample size. The study team will review HIV registers monthly to identify potential participants. All individuals with HIV and/or TB will then be screened by study staff using the AUDIT-C. Those with AUDIT-C scores ≥ 3/4 (women or transgender individuals/men) will be referred by study staff to the study counselor for consent and eligibility determination.

All recruitment activities will take place in a private room to maintain the confidentiality and privacy of the study participants.

##### Randomization procedures: Sequence generation and allocation concealment

Eligible participants will be randomized in a 1:1 ratio upon completion of the baseline evaluation. The study biostatistician, independent of the trial will generate the randomization sequence in permuted blocks with block size 4, and randomization will be stratified by the presence of TB only; HIV only; and TB/HIV co-infection. Allocation will be concealed using sequentially numbered, opaque, sealed envelopes. The study counselor will enroll and assign participants to the interventions

By nature of the interventions being tested, this will be an open-label trial in which neither the subjects, study coordinators, nor interventionists will be blinded to treatment assignment after randomization. However, to decrease potential sources of bias, research assistants, staff abstracting medical records, and data analysts will all be blinded to treatment arm of individual patients.

#### Data Collection and Management

##### Data collection for Aim 1 and Aim 2

###### Research Data Ascertainment:

Research staff blinded to treatment assignment will complete assessments at baseline (prior to randomization), week 2, 4 and 8, and months 3 (end of 4 session intervention), 4, 6, and 12 post-baseline. Data collection visits will be coordinated with scheduled TB or HIV clinic visits. Data collection will include self-report questionnaires (entered on tablets), staff interviews, and biomarker and specimen collection. We include this breadth of measurement strategies to increase the accuracy and scope of our findings (Table 3).

###### Assessment Instrument Selection:

We will use validated instruments to obtain information on alcohol and drug use, mood and quality of life and medication adherence. The baseline research interview is expected to take approximately 1.5 hours. Follow-up data collection will take about 1 hour. Since assessment alone can modify behavior, instruments were selected to balance the need for study data across several outcome domains with the time constraints of data collection. Alcohol measures: Alcohol outcomes, will be measured by a direct alcohol biomarker PEth, a dried blood spot used from the blood sample drawn for other tests. Additionally, drinking days, heavy drinking days, and drinks per drinking day will be measured using the TLFB.

###### PEth:

This direct metabolite of alcohol consumption is formed only in the presence of alcohol, thus it is uniquely specific for recent drinking.(42).(64) PEth is measured in dried blood spots using liquid chromatography with tandem mass spectrometry. The limit of qualification is 8 ng/mL. A value above 50 ng/mL is frequently used to indicate heavy drinking.(64)

###### TLFB:

Using structured interview prompts, the TLFB quantifies alcohol use on each day over the last 30 days. TLFB yields detailed quantity/frequency data including number of abstinent days and drinking days, mean number of drinks/drinking day, and number of heavy (binge) drinking days.(44)

###### Other alcohol measures:

Other measures collected and indicated on Table 3 include measures of alcohol use severity and consequences: MINI International Neuropsychiatric Interview for DSM5)(38); Alcohol Use Disorders Identification Test (AUDIT);(46) the alcohol abstinence self-efficacy scale and an alcohol craving scale.(51, 52, 65)

###### TB Measures:

TB outcomes of medication and appointment adherence will be measured using self-report, biomarkers and clinic records. We use the AIDS Clinical Trial Group (ACTG) adherence questionnaire to measure adherence. In addition, urine and blood samples will be collected to measure urine Isoniazid metabolites and TB drug levels respectively. Retention in care will be measured through clinic registration records and through study records. Sputum will be obtained to determine time to sputum conversion. TB treatment outcomes of cure, treatment failure, and recurrence will be abstracted from the TB treatment card.

###### HIV Measures:

HIV outcomes of medication and appointment adherence and viral suppression will be measured via self-report, clinic registration records, and HIV-RNA measurement, respectively. We will use a self-report 30-day antiretroviral adherence using a visual analog scale,(60) ACTG 4-item adherence questionnaire(61) and pharmacy refill records. Retention in care will be measured through clinic registration records. HIV-RNA and CD4 cell count: HIV-RNA will be drawn as part of the study (as this is not routinely done); CD4 cell count will be abstracted from medical records and drawn if not available.

###### Blood collection:

Blood samples will be collected at baseline, week 4 or 8, 3 months, 6 months and 12 months. The participants will be asked to fast overnight before the blood collection. The blood samples will be used to measure Complete Blood Count (CBC), liver function test, and storage of plasma samples at −80°C for biorepository and future biomarker assessments.

###### Urine samples:

As a secondary measure of TB treatment adherence, we will also measure Isoniazid (INH) metabolites in urine as an objective marker. For people with TB with or without HIV, urine samples will be collected at four time points – baseline, 4 or 8 weeks, 3 months and 6 months. They will be used in a point-of-care test called IsoScreen (GFC Diagnostics) using manufacturer’s directions. This 5-minute dipstick test will assess for TB treatment adherence by measuring INH metabolites in urine. This reflects INH use in the prior 24 hours.

###### TB drug levels:

As a secondary measure of TB treatment outcome, we will also store plasma samples at −80°C for future assessment of TB drug levels (K2 EDTA plasma for Rifampicin and sodium heparin plasma for INH and ethambutol) to study how they are impacted by the intervention. For TB with or without HIV participants, at week 4 or 8 and month 3, blood samples will be collected for pharmacokinetics, 2-hour post TB treatment dose to measure TB drugs.

###### Stool sample:

For participants with TB with or without HIV, stool samples will be collected at four timepoints – baseline, 4 or 8 weeks, 3 months and six months for future assessments on the impact of the intervention on the gut microbiome, and to study the potential mediating role of gut microbiome in the relationship between alcohol use and adverse TB treatment outcomes to complement the parent aims of the study. A pea size scoop of stool size will be self-collected by study participants at home using a kit (NAP-G) with stabilizing transport medium and then will be aliquoted and stored at the study site lab at −80°C until future use. Sample storage: A biorepository will be created to store the plasma and stool samples for future assessments on the impact of the intervention on the microbiome, metabolome and other biomarkers and the potential mediating role of these biomarkers in the relationship between alcohol use and adverse TB treatment outcomes to complement the parent aims of the study. While blood investigations for PK and biomarkers and stool for gut microbiome are important, participants can refuse the collection of samples for ancillary studies and still take part in the main study.

###### Data collection for Aim 3

Following the RE-AIM framework, we will collect qualitative and quantitative data (Table 4) which includes the Mental Health Implementation Science Tool developed for low and middle-income settings. These include assessment of implementation outcomes reach, adoption, feasibility, acceptability, and appropriateness.(66). In addition to these measures on implementation outcomes, we will also include a mixed methods assessment of implementation predictors based on the Consolidated Framework for Implementation Research (CFIR),(67) which will include quantitative items and probes for interviewers to use during qualitative interviews and focus groups.

### Costing measures:

**Figure 3:**
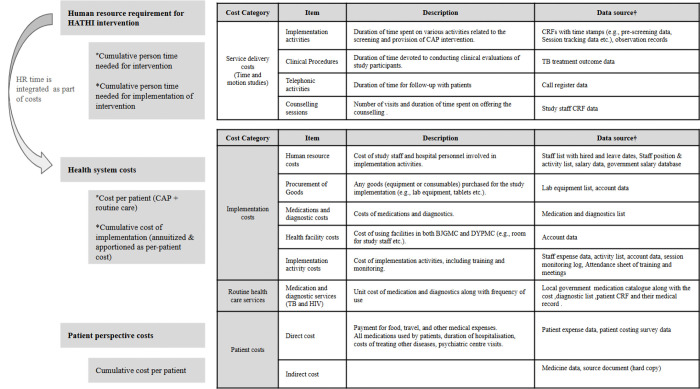
Comprehensive summary of key cost analysis outcomes, data description and sources.

data for economic evaluation will be collected in parallel to the trial to estimate the incremental costs and cost-effectiveness of the HATHI intervention package compared to usual care. Figure 3 provides complete summary of the data collected for the economic evaluation.

#### Time and motion studies:

Data on direct clinical and research staff time committed for implementing and delivering the HATHI intervention will be collected as units of key distinctive service – predefined to distinguish research versus programmatic activities – for each individual participant enrolled in our study from enrolment until end of the follow-up. Time data will be used to assess clinical staff per-patient workloads (i.e. human resource needs to deliver the HATHI intervention) and as an apportionment criterion for fixed costs (e.g. capital assets, overheads) and common costs (resources that are used across more than one distinctive activity in delivering the intervention) in activity-based costing.

#### Health system and implementation costs:

Data on health systems costs will be collected from the perspective of India’s National TB Elimination Program, which is the institution primarily responsible for financing the TB control program. Implementation cost data will be collected using a standardized activity-based time and resource use form following the framework developed by our team(48) and interviews of our research staff, which will be matched to the periodic expenditure log. HATHI intervention costs (other than human resource – assessed using time and motion data and clinical staff pay scales) and other medical service costs (e.g., clinic visit, diagnostics, drugs, etc.) will be collected based on per-patient, service-use frequencies.

#### Patient costs:

Patient’s direct out-of-pocket and indirect costs will be assessed using a modified WHO Patient Cost Survey designed to additionally assess costs associated with alcohol use behavior changes during the study follow-up as well as the patient costs for TB care. Direct costs include costs of medical service fees, transport costs, care-giver costs, and other relevant out-of-pocket costs incurred by patients during intervention participation and TB treatment. Indirect costs account for lost productivity due to alcohol use behavior as well as TB illness, financial coping strategies (e.g., borrowing, selling personal commodities, etc.). As part of this survey, we will also collect patients’ self-reported annual personal and household income and measures of socio-economic status to calculate modified Kuppuswamy scale.(68)

#### Other measures:

All-cause mortality: WHO guidelines will be used for collecting the verbal autopsy for any deaths reported during the study.(63)

#### Data Management:

Data will be captured and stored via REDCap—a secure, HIPAA-compliant, web-based application designed for use in research studies. REDCap features for secure collection and storage of sensitive data include: 1) SSL encryption of data; 2) ability to collect data in real-time to minimize data entry errors; 3) ability to build in real-time data entry validation; and 4) built-in audit trails. Research staff will directly enter data on encrypted laptops/tablets during interactions with patients, except for TLFB interview for alcohol, which will be collected on paper and then double entered into REDCap by study staff. REDCap has ability to check accuracy of double-entered data. Participants can directly enter data via tablets, through the REDCap mobile app.

### Retention

Study staff will complete locator information for all enrolled participants so that they can be tracked if they miss a visit. Prior to the follow up visit study staff will receive a list of expected participants for whom visits are scheduled daily. Participants will be provided with an appointment card with the date of their next scheduled visit and contact details of study staff. Additionally, participants will be reminded by phone call before their scheduled visit to maximize retention. All participants will be advised to contact the study staff if they will not be able to come for their scheduled follow-up visits. In the special instances (e.g. personal or social reasons) where participants are unable to visit the study site within the visit window period, the study staff will carry out a home/telephone/virtual visit. The study sites also have home visitors to contact participants in case they are not reachable by phone.

We have scheduled study and intervention visits to correspond with routine patient clinic visits as much as possible to lessen the burden on participants and improve implementation feasibility if/when this intervention is scaled up beyond our trial. Most participants in this population are expected to have cell phones. Thus, we will set up routine phone calls to increase follow-up. Finally, we have an outreach team and mobile vans to conduct home visits for missed research visits as needed.

Maintaining high rates of follow-up in this study is critical for ascertaining study outcomes. We will provide Rs 350 (USD $4.20) per study visit as compensation for loss of daily wages to the participant. As the study procedure will take 2–3 hours, food will be provided to the participant and others who are accompanying persons. Local transportation will also be paid as actuals by public transport.

### Statistical method

#### Aim 1: Intervention effect on alcohol use

All randomized participants will be included in the primary analysis according to the intervention arm to which they were originally assigned, consistent with the intention-to-treat principle. The primary outcome of the trial is the concentration of PEth measured at 6 months post-randomization. PEth will be analysed as a continuous outcome; distributional assumptions will be assessed, and if the PEth values demonstrate substantial right skewness, an appropriate log transformation will be applied prior to analysis. Given the randomized controlled design, baseline characteristics are expected to be balanced across intervention arms. Nevertheless, we will formally evaluate baseline characteristics by summarizing demographic and clinical variables by study arm using descriptive statistics. The primary analysis will use univariable linear regression to estimate the mean difference in PEth between study arms, incorporating random effects for study site and counsellor to account for potential correlation within these units. If imbalances in baseline characteristics are observed, we will conduct sensitivity analyses to evaluate the impact of these imbalances on treatment effect estimates. Adjusted analyses, including prespecified baseline covariates (e.g., age, gender, religion) and psychosocial and clinical covariates (e.g., depression, smoking, diabetes), will be conducted as supportive and sensitivity analyses to improve precision and to assess the robustness of the primary findings. Participants with missing primary outcome data will not be excluded from the analysis. Instead, missing PEth values will be addressed using prespecified methods, including last observation carried forward and/or multiple imputation, as appropriate. Sensitivity analyses will be performed to compare results across these approaches to evaluate the impact of missing data assumptions on the study conclusions. We will also assess whether the intervention effect varies by treatment intensity (dose), operationalized as the number of intervention sessions attended and the number of booster sessions received. Because factors influencing intervention adherence may also be associated with the outcome, analyses restricted to specific compliance patterns may introduce selection bias. To address this potential bias, we will use inverse probability of censoring weights estimated conditional on baseline and time-varying covariates.

Secondary alcohol-related outcomes include self-reported alcohol consumption assessed using the 30-day TLFB interview. The number of drinking days over the prior 30 days will be analysed as a count outcome, conceptualized as arising from a binomial distribution, Binomial (30, *p*), where *p* represents the proportion of days on which alcohol consumption occurred. The independent effect of the intervention on the proportion of drinking days will be evaluated using adjusted random effects generalized linear models with a logit link function, incorporating random effects for study site and counsellor to account for within group correlation. Differences between intervention arms in the number of self-reported heavy drinking days over the prior 30 days, as measured by the TLFB, will be assessed using multivariable generalized linear random-effects logistic regression models, incorporating random effects for study site and counsellor to account for within group correlation. Intervention effects on other secondary alcohol-related outcomes derived from PEth and AUDIT measures will similarly be evaluated using multivariable random-effects logistic regression models, as appropriate for the outcome distribution. All adverse events (AEs), including serious adverse events (SAEs), and deaths will be monitored and recorded in accordance with the study protocol and established study procedures. AEs and SAEs will be summarized descriptively by study arm using counts and proportions. For comparative analyses between intervention arms, outcomes with binary response distributions will be analysed using multivariable logistic regression models, while count-based AE outcomes will be analysed using multivariable Poisson regression models (or alternative count models if indicated), with appropriate adjustment for clustering and covariates.

#### Aim 2: Intervention effect on TB and HIV clinical outcomes

Composite TB treatment outcomes will be summarized as rates within each intervention arm. Adjusted differences in composite TB outcome rates between intervention arms will be estimated using multivariable Poisson regression models with random effects for study site and counsellor. All-cause mortality over the 12-month follow-up period will be assessed using time-to-event methods. The cumulative mortality function will be estimated using the Kaplan–Meier product-limit estimator, stratified by intervention arm and accounting for clustering by study site and counsellor. Adjusted differences in mortality risk between intervention arms will be estimated using multivariable Cox proportional hazards regression models incorporating random effects for study site and counsellor. Similar survival analysis methods will be applied to evaluate time to sputum-culture conversion among participants with microbiologically confirmed TB.

Differences in HIV-related outcomes, including viral suppression and antiretroviral therapy adherence, will be evaluated using adjusted multivariable logistic regression models, with appropriate specification of random effects and covariates.

Although the study is not explicitly powered to detect differences within individual disease subgroups, all analyses will be conducted stratified by disease cohort, including TB-only, HIV-only, and TB–HIV co-infected participants. This stratified analytic approach will allow for an assessment of the consistency of the BI intervention’s effectiveness across clinically relevant populations. Findings from these subgroup-specific analyses will be interpreted cautiously and considered exploratory in nature. The results are expected to inform hypotheses regarding differential effects and to identify potential gaps requiring targeted investigation in future, adequately powered studies.

#### Aim 3: RE-AIM analyses

Descriptive analyses of quantitative data will be used to investigate the RE-AIM dimensions across stakeholder levels, characterizing the median and distribution of scores. The qualitative data will provide context to the quantitative data and in-depth understanding of barriers and facilitators to program implementation in the local context. Transcripts will be analyzed in an iterative fashion, and concurrent with on-going interviews to allow data to inform queries in subsequent interviews. We will use both deductive and inductive coding for qualitative analysis, deriving codes from the interview guide based on RE-AIM dimensions in addition to identifying emergent themes. Qualitative interviews will be coded by two study team members. The research team will examine these provisional codebooks and create a representative codebook for use in the entire analysis. Inconsistencies will be examined and resolved through consensus. We will merge and integrate quantitative and qualitative data. The qualitative themes will be compared to survey results, combined and jointly displayed to enhance data interpretation.(69, 70).

#### Aim 4: Cost and cost-effectiveness analysis

Cost of the HATHI intervention will be assessed using activity-based costing framework and ingredients approach where resource use and cost data will be assessed based on key HATHI intervention activities. Fixed costs (e.g., HATHI implementation costs) and resources common across multiple intervention activities will be apportion to each activity based on the distribution of direct human resource time committed to each intervention activities. All costs will be assessed in both local (Indian Rupees) and study year United States (US) dollars, with capital assets and fixed costs annuitized using 3% discount rate (varied between 0 and 10% in sensitivity analyses) and relevant expected useful life years.

For patient cost analysis, we will assess the number and percentage of patients avoiding catastrophic cost due to TB as a result of the intervention relative to the control arm. Catastrophic cost will be defined as TB-related patient-incurred cumulative total costs during the observational period exceeding 20% of patients’ annual household income.(71) We will examine the association between catastrophic costs and AE (e.g., treatment failure) using univariable and multivariable analyses generating odds ratios for each variable as risk factor to the catastrophic costs. This will be assessed independently for each study arm and repeated as a combined analysis for difference in proportion of participants experiencing catastrophic costs between the two study arms.

Cost-effectiveness of the intervention will be assessed based on ‘net benefits’ or net-monetary benefits approach,(72) where we will calculate incremental health systems costs per one additional patient successfully completing TB treatment and bench mark this metric against a range of willingness to pay thresholds, starting with the India’s GPD per capital for our analytic year. We will conduct additional analyses on key effectiveness outcomes – incremental cost per participant achieving alcohol reduction and per quality adjusted life years gained – to examine the relationships in the cost-effectiveness outcomes and conclusions across different effectiveness measures.

### Methods in analysis to handle protocol non-adherence and any statistical methods to handle missing data

The sample size calculation incorporates a 10% allowance for loss to follow-up or missing data at the 6-month primary endpoint to preserve 80% statistical power. Given the high rates of attrition typically observed in alcohol intervention trials, we will assess the mechanism of missingness (missing completely at random, missing at random, missing not at random), acknowledging that this is rarely verifiable in practice. Missing data will be addressed using four approaches: last observation carried forward, worst-case scenario imputation (classifying all missing as heavy drinking & using upper limit of PEth detection), multiple imputation with chained equations, and full information maximum likelihood estimation. Auxiliary variables (baseline alcohol use measures, age, TLFB, AUDIT, etc.) will be included in the latter two methods, and sensitivity analyses will be performed across all approaches to evaluate robustness.

### Monitoring

Safety and oversight of the study will be provided by the principal investigators (GC, AG) and the study team in India (NS, AK, RB) and associated Institutional Review Boards (IRB) at BJGMC and DYPMC, 2) a Data Safety and Monitoring Board (DSMB), and 3) the Johns Hopkins Medical Institution’s IRB.

We have established a DSMB consisting of an epidemiologist/physician who implements clinical trials for infectious diseases in India; a clinical psychologist with expertise in alcohol use disorders, alcohol measurement, alcohol clinical trials and behavioral interventions; and a biostatistician. The DSMB is independent of the sponsor and the investigators without competing interests. The DSMB will meet annually and adhoc. There will be no planned interim analyses.

#### Composition of the coordinating center and trial steering committee

The trial will be coordinated through Johns Hopkins University and Johns Hopkins India in partnership with the two Indian clinical sites and investigators at the London School of Hygiene and Tropical Health and University of Washington. The data coordinating center is based in Pune, India and managed by Johns Hopkins India. The coordinating center comprises of experienced trialists, behavioral scientists, study managers, biostatisticians and data managers. The study team, including the PIs and site coordinators, meet every two weeks and review study conduct and recruitment and retention milestones.

#### Harms

At baseline and each follow up visit, participants will receive the CIWA to assess for alcohol withdrawal and they will be asked about withdrawal symptoms. Participants with withdrawal symptoms are referred directly to psychiatry for further evaluation and treatment.

At follow-up visits, all participants will be asked about urgent care/emergency department visits, hospitalizations and reasons for these. All AEs and SAEs are reported to the principal investigators (PI), local medical co-investigators and the local IRBs. Site PIs will be responsible for reporting the SAE to the IRBs of BJGMC and DYPMC for review at respective sites. Any SAE including death or life-threatening condition occurring in the study will be reported to Ethics Committee within 24 hours of site awareness as per Indian National regulatory guidelines and annually to the Johns Hopkins IRB and NIH (if not study related). Any lab values of grade 3 or above per DAIDS AE Grading Table, Corrected Version 2.1, dated July 2017 will be reviewed and reported. SAEs that are deemed to have a probable or definite relationship to study procedures will be promptly reported to study site PIs and to overall PIs who will report to the local IRBs, Johns Hopkins IRB and the NIAAA project officers. In addition, reports will be submitted to the IRBs as part of the annual renewal process and the DSMB, summarizing all AE and SAE and how they were addressed by the study team.

#### Auditing

No independent agency or person will perform an audit. Quality assurance activities are performed by a designated quality assurance officer biannually.

### Ethics and dissemination

#### Protocol amendments

Protocol amendments are reported to the local IRBs, the data monitoring committee and the sponsor. Amendments are also updated in clinical trials.gov.

The following protocol amendments have occurred during the course of this study. We obtained a supplement from the National Institute on Alcohol Abuse and Alcoholism (NIAAA) to add collection and storage of plasma, urine and stool in 2023. We obtained a second supplement from NIAAA in 2024 to add an HIV only arm. The original protocol sought to recruit 450 individuals with TB (n=300) and HIV and TB co-infection (n=150) with unhealthy alcohol use. From the time of grant inception to implementation, HIV and TB co-infection incidence declined due to effective ART. Given lower recruitment of persons with HIV and TB co-infection, we added an HIV only arm, consisting of PWH with unhealthy alcohol use, with the same overall recruitment goal of 450 people. Finally, we changed the primary outcome of the study from self-reported drinking days and heavy drinking days using the TLFB to the alcohol biomarker PEth as recommended by the DSMB on May 30, 2024, due to underreporting alcohol by self-report.

#### Informed Consent Procedure

The informed consent process will be done in person in a private room conducted by study counselors. Study procedures, risk and benefits will be discussed in the local language of choice (Marathi, Hindi and English). The informed consent form includes a description of each of the study visits including survey assessments and biological sample collection; randomization and study condition procedures; remuneration; risks and benefits confidentiality; rights of the participants. Prior to signing the consent form study comprehension will be assessed by a study counselor and participants will be given time to ask any questions they have about the study. While we anticipate the consent process to take 30 minutes, as much time as necessary will be allowed for the consent.

#### Confidentiality

Personal information will be collected for participant tracking purposes and medical record abstraction. We will create unique study IDs for all participants and use these study IDs for all data forms for computer entry and storage. We will report study findings in aggregate only. In addition, all study data are stored in REDCap, which is located on a secure JHU server. All informed consents (PHI) will be stored in the DYPMC and BJGMC data center under double lock and key and only the study team will have access to these files.

## Discussion

Unhealthy alcohol use is a known risk factor for both HIV and Tuberculosis infection acquisition and for poorer HIV and TB treatment outcomes. HATHI,- **H**ybrid trial for **A**lcohol reduction among people with **T**B and **H**IV in **I**ndia, is to our knowledge one of the first hybrid effectiveness-implementation studies testing the effectiveness of an evidence-based behavioral alcohol reduction intervention integrated into TB and HIV clinical care in India. As such, this study will generate new knowledge that will inform delivery of alcohol counseling among people with TB and HIV in these clinical settings

Strengths to this study include the intentional selection of the hybrid design, that allows for the rigor of a randomized controlled trial and the collection of implementation barriers and facilitators of alcohol intervention integration into these clinical settings. Additional strengths include selection of an evidence-based intervention with core components effective in HIV clinical settings and in Indian primary care clinics that has been culturally adapted for people with HIV and TB in India and rigorous quality and fidelity monitoring of behavioral counseling sessions. Finally, use of an alcohol biomarker, PEth, as our primary outcome will overcome challenges of underreported alcohol use due to social desirability.

Despite these strengths, there have been challenges to trial implementation. Specifically, low self-report of alcohol use on the TLFB interview, in contrast to the AUDIT and PEth was noted, resulting in DSMB recommendation to change the primary outcome from self-report to biomarker. Moreover, enrollment of women in the study has been limited, which is likely attributable to the lower prevalence of alcohol use among women in India and potential under-reporting due to the low social acceptability of alcohol use among women in this context. Finally, we have experienced interruptions in recruitment of people with TB at various timepoints in the study due to lack of TB medication availability resulting in reduced screening (April-June 2024) and a TB health staff strike (February 11, 2024-March 5, 2024, and August 18-September 17, 2024).

Notwithstanding these limitations, HATHI will generate new knowledge that will inform delivery of alcohol counseling among people with TB and HIV in these clinical settings. Further, the hybrid design will allow for qualitative exploration of implementation challenges which will directly inform intervention reach and hasten scale up of the intervention should it be effective.

## Supplementary Material

This is a list of supplementary files associated with this preprint. Click to download.

Supplementalmaterial1.docx

## Figures and Tables

**Table 1.** Description of HATHI Intervention Sessions

Session Delivery	Session Content
Session 1: Day of enrollment	• Reasons for and consequences of alcohol use to build individual motivation for change; • Education about standard drinks, lower and high-risk drinking; • Identify moods and situations that trigger use; • Discuss behavioral strategies to manage cravings, high-risk moods, and situations; • Optional goal setting and personal action plan; • Drinking diary for completion between sessions
Session 2: Week 2	• Review alcohol use since the prior session; • Explore challenging moods and situations that lead to drinking, including cravings, social pressure, depression/anxiety; • Develop tailored strategies, including refusal skills, to manage alcohol use; • Develop a personal action plan and discuss tips related to sleep and nutrition.
Session 3: Week 4	• Deepen understanding of how emotions influence alcohol use; • Introduce practical coping strategies such as diaphragmatic breathing exercises, positive self-talk, and engaging in enjoyable, alcohol-free activities to support healthy choices.
Session 4: Week 8	• Relapse prevention: identify thoughts, feelings, and seemingly irrelevant decisions that precede alcohol relapse; • Develop a personalized emergency action plan.
Intervention boosters at months 3, 4, and 6	• Check-in to review progress; • Reinforce previously learned skills and address challenges in alcohol use reduction

**Table 2.** Primary and secondary outcomes for AIM 1 and 2 and their timepoints.

Outcome	3 months	6 months	12 months
**Alcohol(**42,44–46**)**			
PEth quantification	X	X	X
PEth ≥20, 50, 200	X	X	X
Alcohol Use Disorders Identification Test Score	X	X	X
Drinking days in past 30 days (TLFB)	X	X	X
AUDIT score and PEth ≥20, 50, 200	X	X	X
Drinking days in past 30 days and PEth ≥20, 50, 200	X	X	X
**Tuberculosis**			
TB treatment failure, default or mortality (composite)			X
TB treatment failure: smear or culture positive at 5 months		X	X
TB treatment default: interruption of TB treatment for ≥ 2 consecutive months		X	X
All-cause mortality			X
TB treatment adherence		X	
Retention in TB care		X	
**HIV**			
HIV Viral load suppression		X	X
Antiretroviral adherence		X	X
Retention in HIV Care			X

**Table 3.** An overview of assessments and the collection of outcomes.

Evaluations	Screening/Baseline		Weeks			Months			
			2	4	8	3	4	6	12
**Demographics**	**X**								
**Anthropometries (Body mass index) and other vital signs (BP, Pulse)**		**X**		**X**		**X**		**X**	**X**
**Clinical Assessment (occurs routinely at TB and HIV follow-up visits)**		**X**		**X**		**X**		**X**	**X**
**Medical History**		**X**		**X**					
**Alcohol Use**									
30 day Timeline Follow-Back Interview; (43)		**X**				**X**		**X**	**X**
PEth (DBS collection) The blood will be shared from CBC		**X**				**X**		**X**	**X**
AUDIT(46)	**X**			**X**		**X**		**X**	**X**
Alcohol abstinence self-efficacy scale(51)		**X**				**X**		**X**	**X**
Alcohol Craving Scale(52)		**X**				**X**		**X**	**X**
MINI International Neuropsychiatric Interview for DSM5 (38)	**X**								
CIWA(39)		**X**	**X**	**X**	**X**	**X**		**X**	**X**
**Tobacco Use:** Heavy Smoking Index(53)		**X**							
**Drug Use:** WHO ASSIST(54)		**X**				**X**		**X**	**X**
**Depressive/Anxiety Symptoms:** PHQ-8,(55, 56)GAD-7^;^(57)		**X**				**X**		**X**	**X**
**Quality of Life:** WHO QOL-BREF;(58) Oslo Socials support,(59) and EQ-5D(50)		**X**				**X**		**X**	**X**
**Patient Costing Survey**		**X**			**X**			**X**	**X**
**Diet**		**X**		**X**		**X**		**X**	
**HIV Outcomes** *									
Adherence: Visual Analog Scale/ACTG measure(60, 61)		**X**				**X**	**X** ☒	**X**	**X**
CD4+/HIV RNA		**X**						**X**	**X**
Retention In Care (Clinic records)									**X**
**TB Outcomes** ^#^									
ACTG adherence questionnaire(62)			**X**	**X**	**X**	**X**	**X** ☒	**X**	
TB Treatment outcome (cure)								**X**	**X**
Chest X-Ray		**X**						**X**	
PTB Culture		**X**	**X**	**X**	**X**			**X**	
AFB Smear		**X**	**X**	**X**	**X**			**X**	
GeneXpert		**X**							
Retention in Care and mortality (Clinic records) (63)								**X**	**X**
**Informational, Motivational, Behavioral Skills Assessment**									
Readiness and confidence in ability to change alcohol use; (Readiness Ruler (Visual Analog Scale))		**X**				**X**		**X**	**X**
**Sample collection**									
Fasting Blood Collection for metabolomic assessments^#^ (Blood volume – 4 ml at each collection)		**X**		**X**		**X**		**X**	
PK assessment – post 2 hr of TB treatment dose^#^ (Blood volume – 8 ml at each collection)				**X**		**X**			
CBC (Blood volume – 1 ml at each collection)		**X**				**X**		**X**	**X**
LFT (Blood volume – 2 ml at each collection)		**X**				**X**		**X**	**X**
Urine^#^		**X**		**X**		**X**		**X**	
Stool Collection^#^		**X**		**X**		**X**		**X**	

* Applicable to only HIV-positive participants

☒ Telephonic visit, if the participant is unable to visit the site.

# Applicable to TB and HIV-TB coinfected participants

**Table 4.** Sample of Quantitative and Qualitative Implementation Measures

Outcomes	Quantitative Measures	Sampling of qualitative interview content
Reach	1. Number of individuals eligible for RCT 2. Proportion of individuals eligible who enrolled 3. Characteristics of eligible participants that enroll compared to those who do not 4. JHU Implementation Measures (counselors)	1. Reasons for enrolling or not enrolling (patient) (e.g. structural (transportation); social/personal (stigma/family), patient preferences for treatment (where to receive treatment) 2. What could be done to increase participation (patient, counselors, clinic/organizational staff)
Effectiveness	1. RCT effectiveness outcomes described in Aims 1 & 2. Including by intervention dose received (number of HATHI sessions attended, number of booster received)	1. Perceptions of why intervention did or did not work (patients and staff) 2. Barriers/Facilitators to use of individual intervention components (patients) 3. Barriers/Facilitators to intervention participation (patients)
Adoption*	1. JHU Implementation measures (patients, counselors, clinic/organizational staff) on adoption, feasibility, acceptability, and appropriateness	1. Barriers to adoption (screening, intervention uptake), steps to enhance uptake; perceived need for intervention, perceived fit in current care structure (counselors, medical/clinic staff, organizational/policy leaders)
Implementation	1. Intervention fidelity: Audiorecordings of intervention sessions with fidelity check lists 2. Number of intervention sessions and boosters delivered 3. Number of training and retraining in intervention required 4. Intervention costs	1. How will screening and intervention need to be adapted; challenges to implementing the intervention per protocol; perceptions of training (counselors) 2. Advantages/disadvantages to delivery in TB/HIV setting instead of traditional alcohol treatment and costs, resources and burden of intervention (counselors, medical/clinic staff, organizational/policy leaders)
